# Supplementary material for: Complexome profiling of the Chlamydomonas psb28 mutant reveals TEF5 as an early PSII assembly factor
Source: Plant Cell. 2025 Mar 17;37(6):koaf055. doi: 10.1093/plcell/koaf055 (PMC12164590; doi:10.1093/plcell/koaf055)
Supplement: koaf055_Supplementary_Data [file koaf055_supplementary_data.zip › psb28_interactive.zip!/plots/intensity/qualityPlots/peptides/HSP70B_Cre06.g250100.t1.2.html]

### Cre06.g250100.t1.2

Trivial name: HSP70B  
SeqCoverage [median]: 30.30  
Mass: 71.95 kDa  
  
 

| Sequence | MissedCleavage | Mass [Da] | wtIntensity | muIntensity | Score |
| --- | --- | --- | --- | --- | --- |
| AALPADDAEATK | 0 | 1171.57 | 2462.32 | 3108.42 | 102.06 |
| GLAVSVR | 0 | 700.42 | 0.00 | 49192.27 | 94.67 |
| IINEPTAASLAYGFDKK | 1 | 1836.96 | 5645.42 | 22989.16 | 103.61 |
| IPAVQEIVR | 0 | 1023.61 | 4427.17 | 9696.16 | 88.37 |
| ITGASTLDKGDVER | 1 | 1460.75 | 9719.87 | 19463.34 | 89.99 |
| IVDFLADDFKK | 1 | 1309.69 | 12512.39 | 26109.88 | 90.65 |
| KLTEDAAK | 1 | 874.48 | 479.27 | 2624.19 | 126.24 |
| LSISDIQEVILVGGSTR | 0 | 1785.98 | 1317.89 | 0.00 | 75.44 |
| LTEAAEK | 0 | 760.40 | 687.09 | 2343.97 | 105.39 |
| MSEVGSESTQVPYR | 0 | 1568.71 | 1978.53 | 2645.19 | 153.95 |
| NQAETMVYQTEK | 0 | 1440.66 | 1904.96 | 2689.13 | 116.77 |
| NTTLPTSK | 0 | 860.46 | 671.25 | 3730.07 | 89.75 |
| QAVVNPENTFFSVK | 0 | 1578.80 | 2405.38 | 0.00 | 93.10 |
| SEGIDLR | 0 | 788.40 | 1095.24 | 0.00 | 73.04 |
| TTPSVVAFTK | 0 | 1049.58 | 1479.73 | 4503.55 | 87.31 |
| VIEDGGNVK | 0 | 929.48 | 1114.25 | 620.91 | 75.11 |
|  |  |  | 47900.7 | 149716.2 |  |
